# Generative artificial intelligence enables the generation of bone scintigraphy images and improves generalization of deep learning models in data-constrained environments

**DOI:** 10.1007/s00259-025-07091-8

**Published:** 2025-01-29

**Authors:** David Haberl, Jing Ning, Kilian Kluge, Katarina Kumpf, Josef Yu, Zewen Jiang, Claudia Constantino, Alice Monaci, Maria Starace, Alexander R. Haug, Raffaella Calabretta, Luca Camoni, Francesco Bertagna, Katharina Mascherbauer, Felix Hofer, Domenico Albano, Roberto Sciagra, Francisco Oliveira, Durval Costa, Christian Nitsche, Marcus Hacker, Clemens P. Spielvogel

**Affiliations:** 1 Department of Biomedical Imaging and Image-guided Therapy, Division of Nuclear Medicine, Medical University of Vienna, Spitalgasse 23, Vienna, 1090 Austria; 2 Christian Doppler Laboratory for Applied Metabolomics, Medical University of Vienna, Vienna, Austria; 3 IT4Science, IT Services & Strategic Information Management, Medical University of Vienna, Vienna, Austria; 4 Nuclear Medicine-Radiopharmacology, Champalimaud Clinical Centre, Champalimaud Foundation, Lisbon, Portugal; 5 Department of Experimental and Clinical Biomedical Sciences, Nuclear Medicine Unit, University of Florence, Florence, Italy; 6 ASST Spedali Civili of Brescia, Università degli Studi di Brescia, Brescia, Italy; 7 Department of Internal Medicine II, Division of Cardiology, Medical University of Vienna, Vienna, Austria

**Keywords:** Synthetic data, Generative, Artificial intelligence, Bone scintigraphy, Multicenter

## Abstract

**Purpose:**

Advancements of deep learning in medical imaging are often constrained by the limited availability of large, annotated datasets, resulting in underperforming models when deployed under real-world conditions. This study investigated a generative artificial intelligence (AI) approach to create synthetic medical images taking the example of bone scintigraphy scans, to increase the data diversity of small-scale datasets for more effective model training and improved generalization.

**Methods:**

We trained a generative model on ^99m^Tc-bone scintigraphy scans from 9,170 patients in one center to generate high-quality and fully anonymized annotated scans of patients representing two distinct disease patterns: abnormal uptake indicative of (i) bone metastases and (ii) cardiac uptake indicative of cardiac amyloidosis. A blinded reader study was performed to assess the clinical validity and quality of the generated data. We investigated the added value of the generated data by augmenting an independent small single-center dataset with synthetic data and by training a deep learning model to detect abnormal uptake in a downstream classification task. We tested this model on 7,472 scans from 6,448 patients across four external sites in a cross-tracer and cross-scanner setting and associated the resulting model predictions with clinical outcomes.

**Results:**

The clinical value and high quality of the synthetic imaging data were confirmed by four readers, who were unable to distinguish synthetic scans from real scans (average accuracy: 0.48% [95% CI 0.46–0.51]), disagreeing in 239 (60%) of 400 cases (Fleiss’ kappa: 0.18). Adding synthetic data to the training set improved model performance by a mean (± SD) of 33(± 10)% AUC (*p* < 0.0001) for detecting abnormal uptake indicative of bone metastases and by 5(± 4)% AUC (*p* < 0.0001) for detecting uptake indicative of cardiac amyloidosis across both internal and external testing cohorts, compared to models without synthetic training data. Patients with predicted abnormal uptake had adverse clinical outcomes (log-rank: *p* < 0.0001).

**Conclusions:**

Generative AI enables the targeted generation of bone scintigraphy images representing different clinical conditions. Our findings point to the potential of synthetic data to overcome challenges in data sharing and in developing reliable and prognostic deep learning models in data-limited environments.

**Graphical abstract:**

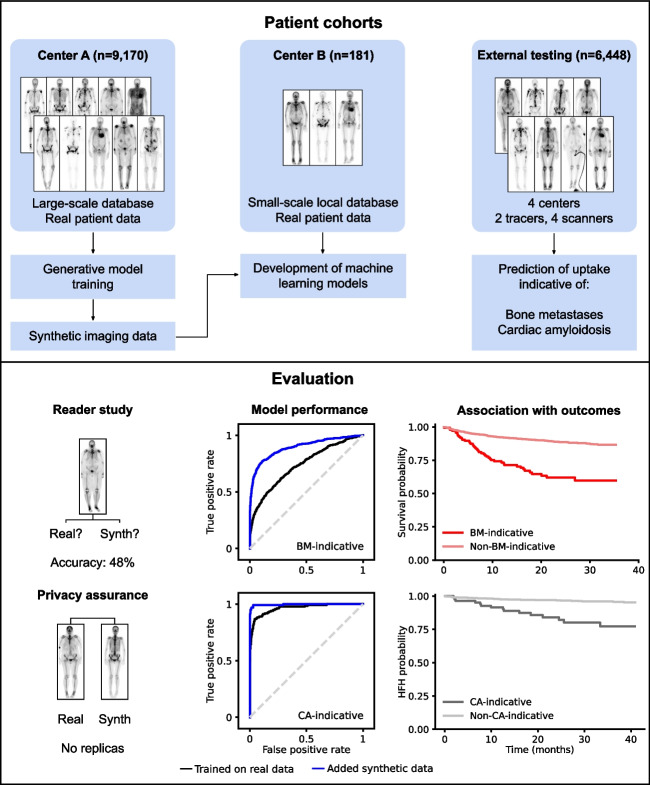

**Supplementary Information:**

The online version contains supplementary material available at 10.1007/s00259-025-07091-8.

## Introduction

The rise of artificial intelligence (AI) has introduced a paradigm shift in medical imaging. Deep neural networks have shown promising results in the accurate diagnosis of diseases [1, 2], segmentation of anatomical structures (*3*), prediction of patient prognosis [4, 5], and treatment response evaluation [6], with increasing numbers of AI-related procedures applied as part of clinical routine [7]. Moreover, AI is being studied for image reconstruction and formation tasks, such as scatter and attenuation correction, with the aim of improving image quality, reducing noise, achieving faster processing times, and potentially lowering radiation doses for patients [8].

This advancement, however, is restricted by the availability of large, annotated datasets, as substantial quantities of data are required to train deep learning models effectively. The collection of such large datasets is costly and laborious. Hence, it poses a significant challenge, particularly in the medical field, necessitating the organization of extensive multicenter studies to assemble large, representative, and annotated patient cohorts. Yet, the endeavor of pooling data across different institutions is difficult, primarily due to the complexities surrounding data sharing and patient privacy concerns inherent to healthcare research. These difficulties hinder models from generalizing to unseen data and hamper their clinical translation.

Synthetic data generation provides a promising alternative to complement training datasets and increase research scale [9]. Recent advances in generative AI have shown promising results in synthesizing medical images such as chest X-rays [10–12]. However, these studies did not explore the applicability of synthetic image generation to molecular imaging, where the fundamentals of image acquisition and clinical indication differ. Hence, pre-existing findings from these studies cannot be adopted to other imaging domains without thorough investigation.

Bone scintigraphy plays an important role in the workup of various diseases and conditions such as osseous metastases, cardiac amyloidosis, Paget’s disease, radiographically occult injury, osteomyelitis, and the assessment for prosthesis infection or loosening. Most commonly, patients with solid tumors at risk for osteoblastic bone metastases are being imaged. The five-year survival rates after de *novo bone* metastasis diagnosis have been estimated in 2022 as 22% for prostate, 23% for breast, 2% for lung, and 7% for renal cancer [13]. While promising new treatments have emerged in recent years, potentially improving survival rates, such as [^177^Lu]Lu-PSMA radioligand therapy [14] or targeted radionuclide therapy with the alpha emitter Radium-223 for metastatic prostate cancer [15], the prognosis for patients with bone metastases remains poor. Thus, the identification of abnormal uptake indicative of bone metastases is of great importance and enables interventions to reduce pain, maintain mobility, and improve quality of life. Moreover, bone scintigraphy has recently emerged as a valuable tool for identifying cardiac amyloidosis. Transthyretin cardiac amyloidosis (ATTR-CA) has lately gained significant interest due to recent advances in diagnostics [16], treatment [17], and increased disease awareness. Scintigraphy imaging with bone-avid tracers enables the non-invasive diagnosis of ATTR-CA through visual assessment. Early treatment in ATTR-CA has been associated with improved survival [18]. Accurate and timely diagnosis via scintigraphy can therefore play a crucial role in initiating life-saving ATTR treatment.

This study aimed to generate clinically meaningful synthetic bone scintigraphy imaging data to enrich small-scale datasets and to improve the development of deep learning models in settings with limited data. To demonstrate the generalizability of this approach, we focused on generating images representing two clinical conditions: (i) tracer uptake indicative of bone metastases and (ii) cardiac uptake indicative of cardiac amyloidosis.

## Materials and methods

### Study design and patient cohorts

**Fig. 1:**
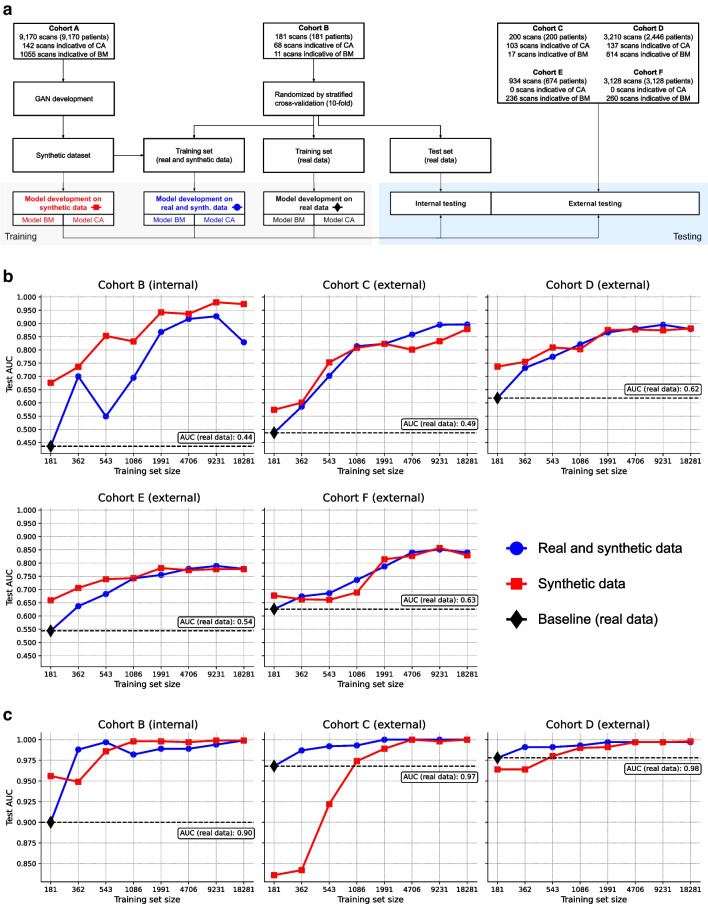
**a** Study design. **b** and **c** Model performances for different training set sizes and real-to-synthetic data ratios for (**b**) the prediction of uptake indicative of bone metastases and (**c**) the prediction of uptake indicative of cardiac amyloidosis. For the latter in (**c**), no AUC could be calculated for the external validation cohorts E-F as there were no cases with CA-indicative uptake

This study included 15,799 patients (16,823 scans) from five different sites (Vienna General Hospital, Austria; ASST Spedali Civili of Brescia, Italy; Careggi University Hospital, Italy; Champalimaud Foundation, Portugal; and West China Hospital, China) who underwent clinically indicated whole-body ^99m^Tc-scintigraphy imaging with bone-avid tracers (^99m^Tc-DPD and ^99m^Tc-HMDP) between 2010 and 2023. Of those, 9,170 patients from one center (Vienna General Hospital, collected between 2010 and 2020, Cohort A) served as the development dataset for the training and validation of a generative model. To that end, we created three different downstream classification scenarios to demonstrate how synthetic data can overcome data-sharing barriers by complementing small single-center databases. In the first scenario, we trained a deep learning model on all available local training data from a single center (181 patients from the ASST Spedali Civili of Brescia collected between 2016 and 2022, Cohort B). This setting simulated an extreme scenario where only limited data is available, representing a small community hospital with a low patient throughput. While 181 might be a low number of patients for deep learning applications, the purpose of this experiment was to demonstrate that even with a real-world dataset from a small-scale center, models can still be developed effectively with the aid of synthetic data. The performance of this model served as the baseline for subsequent experiments. In the second scenario, we evaluated a setting where transferring real data between two or more institutions is challenging. Here, we complemented the small single-center dataset with synthetic images to determine if the model’s performance can be improved compared to the baseline. Thirdly, we trained a model with only synthetic data to simulate an extreme case where no real data is available and to test if synthetic data itself can be used to build a reliable model. In all three scenarios, classification tasks were performed for two clinical targets: (i) tracer uptake indicative of bone metastases (BM-indicative) and (ii) cardiac uptake indicative of cardiac amyloidosis (CA-indicative). The remaining four cohorts comprised 6,448 patients (7,472 scans) and included 200 patients (200 scans) from the Careggi University Hospital (Cohort C), 2,446 patients (3,210 scans) from the Vienna General Hospital (collected between 2020 and 2023, Cohort D), 674 patients (934 scans) from the Champalimaud Foundation (Cohort E), and 3,128 patients (3,128 scans) from the West China Hospital (Cohort F). These four cohorts were held out for independent and external cross-tracer and cross-scanner testing and to associate the model predictions with clinical outcomes. The study design is outlined in Fig. 1A.

**Fig. 2:**
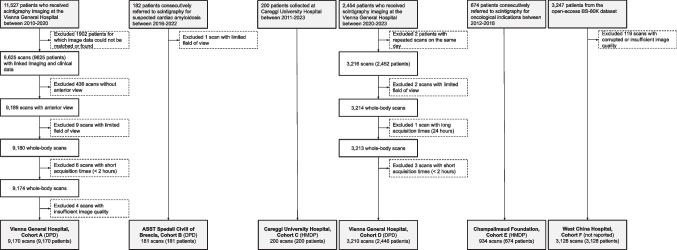
Cohort Flow Diagram

An all-comer recruitment strategy was employed to ensure real-life routine conditions. Hence, inclusion criteria were designed to be as inclusive as possible for the patient cohort from the Vienna General Hospital, aiming to acquire all consecutive patients referred for whole-body ^99m^Tc-scintigraphy (all-comers) between 2010 and 2023. For the patient cohort from Careggi University Hospital, an equal number of cases with and without cardiac amyloidosis-indicative uptake were selected. Details of the patient cohort from the West China Hospital (open-access data) have been published elsewhere [19]. Exclusion criteria were image acquisition at less than 2 h after radiotracer injection following the recommendations of current clinical guidelines [20, 21] and if the image quality was insufficient for clinical usage. A cohort flow diagram is shown in Fig. 2.

This study was granted ethical approval by the institutional review board of the Medical University of Vienna (1376/2024). The requirement to obtain informed consent was waived.

### Imaging and ground truth annotation

All scans were acquired after at least 2 h post-injection [20, 21]. An overview of the scanners and tracers utilized for each cohort is presented in Supplemental Table 1. The ground truth annotation of BM-indicative tracer uptake was performed for the purpose of this study by a nuclear medicine physician, who had access to the image and the clinical indication of the scan if available. The physician scored images as positive if (i) they showed abnormal tracer uptake that may indicate bone metastases and (ii) if further imaging or bioptic examinations would be recommended. The same procedure was performed for all cohorts except for Cohort E. There, ground truths were established based on the clinical reports of the scans. For CA-indicative uptake, ground truth annotation was derived from the consensus reading of at least three independent experts according to the Perugini grading scale and current clinical recommendations [16, 21]. In alignment with the clinical guidelines, scans were considered CA-indicative if rated with Perugini grade 2 or 3. Patient cohort characteristics are presented in Table 1.

**Table 1:** Patient cohort characteristics

Variable	Vienna General Hospital (Cohort A)	Vienna General Hospital (Cohort D)	ASST Spedali Civili of Brescia (Cohort B)	Careggi University Hospital (Cohort C)	Champalimaud Foundation (Cohort E)	West China Hospital* (Cohort F)
No. of patients	9,170	2,446	181	200	674	3,128
No. of scans	9,170	3,210	181	200	934	3,128
Age (years)	61.8 (15.7)	64.1 (15.9)	76.4 (9.9)	78.1 (8.3)	63.6 (12.4)	-
Missing	0 (0%)	0 (0%)	0 (0%)	0 (0%)	0 (0%)	3,128 (100%)
Sex						
Male	3326 (36%)	1325 (54%)	136 (75%)	154 (77%)	277 (41%)	-
Female	5844 (64%)	1121 (46%)	39 (22%)	46 (23%)	397 (59%)	-
Missing	0 (0%)	0 (0%)	6 (3%)	0 (0%)	0 (0%)	3,128 (100%)
Cardiac amyloidosis						
Indicative	142 (2%)	137 (6%)	68 (38%)	103 (52%)	0 (0%)	0 (0%)
Non-indicative	9,028 (98%)	2,309 (94%)	113 (62%)	97 (48%)	674 (100%)	3,128 (100%)
Bone metastases						
Indicative	1,055 (12%)	614 (25%)	11 (6%)	17 (9%)	110 (16%)	260 (8%)
Non-indicative	8,115 (88%)	1,832 (75%)	170 (94%)	183 (91%)	564 (84%)	2,868 (92%)

*Age and sex were not made available on a patient level

### Image preprocessing

All scans were resampled to a target spacing of 2 × 2 mm^2^. In contrast to existing studies [10, 11], original image resolutions were kept at 1024 × 256 and not downsampled (i.e. reduced in resolution), thereby maintaining clinical applicability. High-resolution images are critical in clinical practice as they preserve fine details that are essential for accurate diagnosis and interpretation. By using the original resolution, we aimed to ensure that the generated synthetic images are more similar real-world clinical images where high-resolution images are used. Additionally, downsampling can result in the loss of important diagnostic information, which might negatively impact the model’s performance and its ability to generalize in real-world clinical settings. Image intensities were normalized using the 99.5 percentile intensity values per scan for clipping. Only the anterior views of the bone scintigraphy scans were utilized.

### Generative model

We used a generative model (StyleGAN2) to create synthetic bone scintigraphy images [22]. The model was trained from scratch using the clinical targets as conditional variables, enabling us to synthesize images with specific conditions. This was achieved by providing the ground truth label of an image to the network during training (Supplemental Fig. 3). This procedure allowed the network to learn how to generate synthetic images with different attributes, i.e., an image representing a patient with BM-indicative or CA-indicative tracer uptake. During inference, the model can then be tasked to generate synthetic images with different labels. Additionally, we trained a convolutional neural network (CNN) on the same training data and applied it to the synthetic images to ensure the correctness of the generated images. In this additional data curation step, a synthetic image was kept only if the CNN prediction matched the output of the generative model. We used the Fréchet inception distance (FID) as the primary metric for model selection. Details about the network architecture and training are provided in Supplemental Material A.

### Qualitative assessment

The uniform manifold approximation and projection (UMAP) method [23] was used to create a two-dimensional embedding (2D scatterplot) to visualize the training data distribution consisting of real patient images and the inference data distribution consisting of synthetically generated images. The alignment of the two distributions reflects an approximation of whether the generative model has successfully learned to model the underlying real data distribution, i.e., to generate highly realistic synthetic images with similar attributes, e.g., an image showing a patient with BM-indicative uptake.

### Reader study

The quality and clinical value of the generated images were evaluated in two separate blinded reader studies, one for each clinical target. For each study, 200 scans were randomly selected, comprising 50 real positive, 50 real negative, 50 synthetic positive, and 50 synthetic negative images. All readers were blinded to the distribution of real and synthetic images and the number of positive and negative cases to avoid bias in their assessment. Four different physicians were independently asked to score whether they thought that a given image was real or synthetic. The images were presented individually in a randomized order. An explanation had to be provided if the reader marked an image as synthetic. There were no time constraints for this task.

### Privacy assurance assessment

Image similarity metrics between each synthetic and real image were calculated to ensure that the generative model is not merely replicating images from the training set. The pixel-wise difference between each synthetic image and training sample in the development dataset was measured. To account for spatial variations (e.g., the model could copy a real patient but shifted by a few pixels), we extracted image features and performed a nearest neighbors’ analysis (Supplemental Material B).

### Deep learning model and downstream classification

Two deep learning models (one for each classification task) were built to evaluate the clinical value of the synthesized data in a downstream classification task. Both models were trained in a stratified 10-fold cross-validation scheme using a dedicated training, validation, and test set. Model inference on external holdout test sets was performed using an ensemble of all ten cross-validated models, with predictions determined by majority vote. A scan was predicted as positive if the predicted probability was greater than or equal to 0.5. Standard data augmentations (rotations, zooms, additive noise, shifted and scaled intensities, smoothing) and random oversampling of the minority class were performed by default for all experiments on the training set. Both models were pre-trained on the ImageNet database [24]. Details about the training procedure are described in the Supplemental Material C. To further understand the impact of synthetic data on the model performance, we performed an augmentation study for different amounts of added synthetic data. We augmented the baseline training dataset by real-to-synthetic data ratios of 1:2 (*n* = 543), 1:5 (*n* = 1,086), 1:10 (*n* = 1,991), 1:25 (*n* = 4,706), 1:50 (*n* = 9,231), and 1:100 (*n* = 18,281), while keeping the relative class distribution constant to simulate realistic prevalences of the disease.

### Patient outcome data

We assessed the model predictions (i.e., predicted BM-/CA-indicative uptake) for their association with clinical endpoints to ensure the validity and clinical value of the deep learning models. This is important because a model’s ability to accurately predict clinical outcomes directly reflects its potential utility in real-world clinical decision-making, ensuring that the model is not only technically valid but also relevant for patient care. The endpoint for BM-indicative uptake was all-cause mortality. Heart failure-associated hospitalization (HFH) was the endpoint for analyzing patients with predicted CA-indicative uptake. Data on patient outcomes were collected for Cohort D. For both endpoints, the day of scintigraphy was used as the starting date. The end of the follow-up period was July 14, 2023, and December 31, 2023, for all-cause mortality and HF-associated hospitalization, respectively. Data on all-cause mortality was acquired via the nationwide Austrian Death Registry. Heart failure-associated hospitalizations were determined from three sources, i.e., patient records of the Medical University of Vienna, the Vienna-Health-Association database, and the nationwide electronic health records.

### Statistical analyses

Continuous data are presented as means with standard deviations (SD) or medians with interquartile ranges (IQR). Categorical variables are shown as numbers and percentages. Reader study performance was measured using accuracy, and interrater variability was assessed with Fleiss’ Kappa. The primary metric for downstream classification was the area under the receiver-operating characteristic curve (AUC) with a fixed operating point of 0.5. The DeLong test was used to compare the AUCs of different models. Event rates between patient groups were calculated using Kaplan-Meier estimators and the log-rank test. Only the first scan was included in the outcome analysis for patients with multiple scans. Initial and follow-up scans were used for downstream classification performance. Cox proportional hazards models were used for outcome analysis, with multivariate adjustment for known available confounders and demographic factors (age and sex). Confidence intervals for deep learning performance were calculated using 1,000 bootstrapped samples. P-values ≤ 0.05 were considered statistically significant. This study was performed in accordance with the CLAIM guidelines [16].

## Results

### Generation of synthetic bone scintigraphy scans

**Fig. 3:**
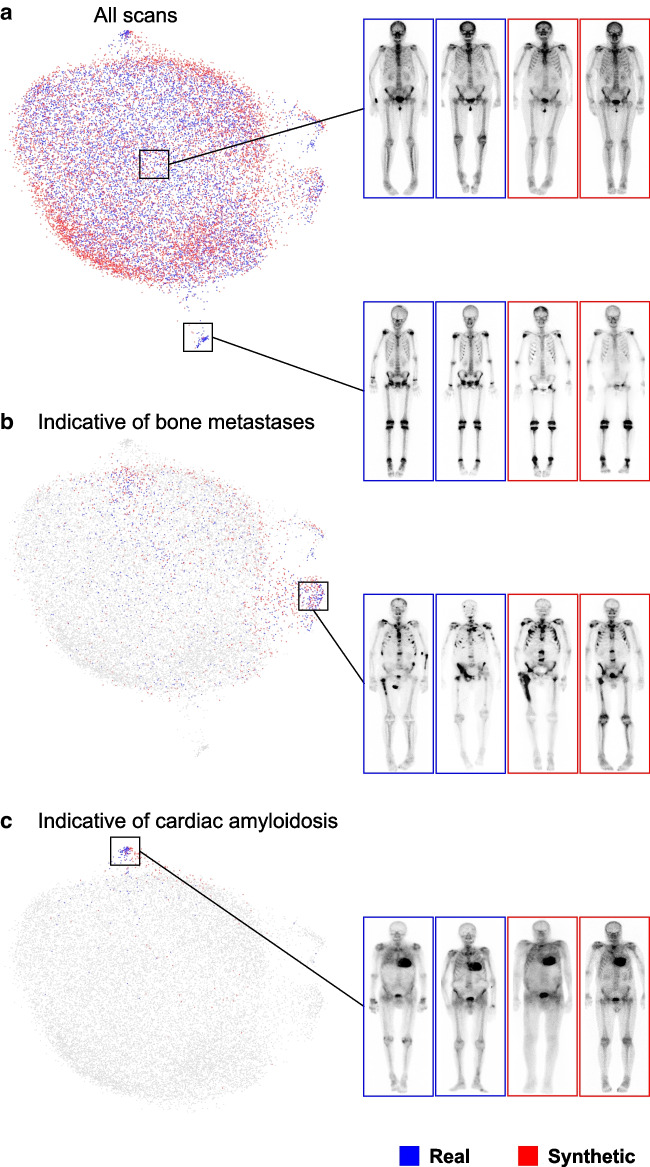
Visualization of the training (blue, real images) and inference data (red, synthetic images), based on a two-dimensional uniform manifold approximation and projection (UMAP) embedding, where each dot represents an image. Real and synthetic data distributions are shown for (**a**) all scans, **b** scans indicative of bone metastases, and (**c**) scans indicative of cardiac amyloidosis. The number of positive and negative cases of the synthetic data was set to the training data, reflecting realistic disease prevalences

A visualization of the training dataset and the synthesized instances, including example images, is presented in Fig. 3. The alignment of the real and synthetic data distribution suggests that the generative model successfully learned to synthesize images from the underlying training distribution, irrespective of the clinical condition. Details about the model selection are in Supplemental Fig. 1. A dataset of representative synthetic images is publicly available (10.5281/zenodo.13275306).

### Expert readers cannot differentiate between real and synthetic scans

The four readers could not distinguish synthetic scans from real scans and had an average accuracy of 0.477 (95% CI 0.446–0.506) among patients with or without BM-indicative uptake, and 0.490 (0.459–0.522) for CA-indicative uptake, respectively. The four readers disagreed in 239 (60%) of 400 cases (inter-observer variability: Fleiss’ kappa 0.18, Table 2). Overall, all four readers correctly identified 6 (1.5%) out of 400 scans as synthetic, of which four showed BM-indicative uptake and two CA-indicative uptake. Common characteristics for correctly identifying these scans, as specified by the readers, included unrealistic uptake at several anatomical sites and unnatural shapes of anatomical structures (e.g., deformed feet).

**Table 2:** Results of the reader study in discerning synthetic scans from real scans

Task	Cohort (scans)	Reader	Accuracy	95% CI	Fleiss’ kappa
Uptake indicative of BM	All cases (*n* = 200)	R1	0.480	0.425–0.530	
		R2	0.466	0.395–0.535	
		R3	0.481	0.425–0.530	
		R4	0.483	0.415–0.545	0.16
	BM-indicative (*n* = 100)	R1	0.490	0.410–0.570	
		R2	0.469	0.370–0.570	
		R3	0.491	0.400–0.570	
		R4	0.490	0.400–0.580	0.20
	Non-BM-indicative (*n* = 100)	R1	0.470	0.400–0.540	
		R2	0.459	0.370–0.540	
		R3	0.471	0.420–0.510	
		R4	0.481	0.390–0.570	0.08
Uptake indicative of CA	All cases (*n* = 200)	R1	0.474	0.425–0.520	
		R2	0.468	0.400–0.540	
		R3	0.454	0.400–0.510	
		R4	0.561	0.490–0.630	0.20
	CA-indicative (*n* = 100)	R1	0.470	0.390–0.550	
		R2	0.450	0.360–0.540	
		R3	0.408	0.330–0.490	
		R4	0.658	0.570–0.740	0.04
	Non-CA-indicative (*n* = 100)	R1	0.481	0.430–0.530	
		R2	0.490	0.400–0.570	
		R3	0.500	0.450–0.550	
		R4	0.459	0.410–0.510	0.12

*BM *Bone metastases, *CA*  Cardiac amyloidosis, *CI * Confidence Interval, *R* Reader

### Synthetic scans are not merely replicas of the training data

**Fig. 4:**
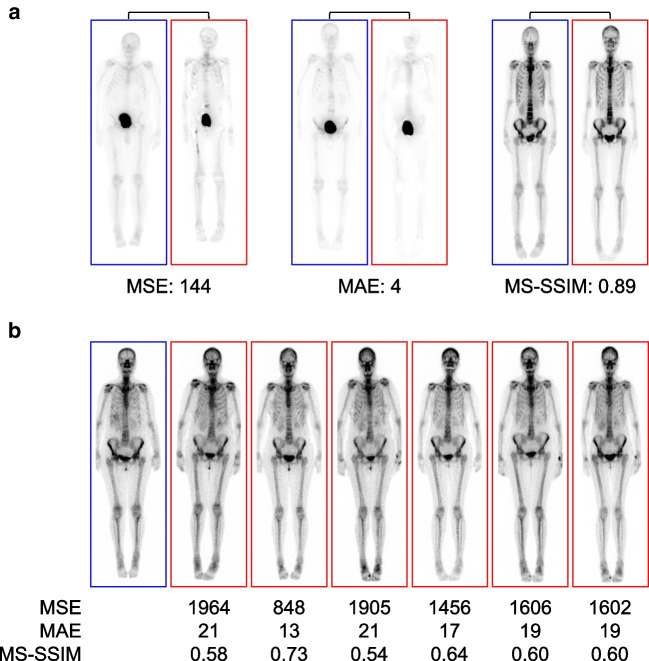
Most similar cases among the training dataset (blue) and the generated synthetic images (red). **a** Scans with the overall lowest mean squared error (MSE), mean absolute error (MAE), and the highest multi-scale structural similarity index measure (MS-SSIM). **b** Real patient scan (blue) together with his six nearest neighbors across the entire dataset (red)

The image similarity analysis revealed no exact replicas between the real and synthesized images. The most similar pairs had a mean squared error (MSE) of 144, mean absolute error (MAE) of 4, and multi-scale structural similarity index (MS-SSIM) of 0.89 (Fig. 4A). In Fig. 4B, we show a real scan from a patient along with the six most similar synthetic images (nearest neighbors) from the entire dataset.

### Diagnostic performances of models trained on real and synthetic data

The baseline model trained on all available local (real) data from a small single-center dataset (*n* = 181 scans, Cohort B) only achieved an internal cross-validated AUC of 0.436 (95% CI 0.251–0.639) for the BM-indicative classification task. The performance of this baseline model on external testing was in line, with an AUC of 0.487 (0.333–0.647) for Cohort C, an AUC of 0.618 (0.590–0.644) for Cohort D, an AUC of 0.544 (0.500–0.592) for Cohort E, and an AUC of 0.626 (0.590–0.662) for Cohort F. Adding the same number of synthetic images to the training set (i.e., *n* = 181 synthetic scans resulting in a real-to-synthetic data ratio of 1:1 or *n* = 362 scans) improved the model performance by a mean (± SD) of 12(± 7)% AUC (*p* = 0.8239) over all test cohorts. The results are shown in Fig. 1B (blue line). With increasing training samples, the model performance substantially improved and eventually converged at a real-to-synthetic data ratio of 1:50 (*n* = 9,231), resulting in an absolute improvement of 33(± 10)% AUC (*p* < 0.0001) on average across both internal and external testing cohorts. The results for this mixed (real and synthetic data) training scheme were benchmarked against purely synthetic training, with the same number of training data but only synthetic scans. The performances of purely synthetically trained models are shown in Fig. 1B (red line). Similar performances were observed except for the internal test set, where the synthetic training outperformed the mixed training scheme. A comprehensive overview of the model performances for each data ratio is shown in Supplemental Table 2.

A comparable trend was observed for the second classification task (Fig. 1C). The cross-validated baseline performance of the CA-indicative prediction model was AUC 0.900 (0.850–0.942) for the internal test set (Cohort B), and AUC 0.968 (0.946–0.986) for Cohort C, and AUC 0.978 (0.961–0.992) for Cohort D on external testing. Since there were no CA-indicative positive cases in Cohort E-F, no AUC could be calculated. Adding synthetic images to the training improved the test AUC in all centers. The highest increase of 5(± 4)% AUC (*p* < 0.0001) on average over all test cohorts was observed for a real-to-synthetic data ratio of 1:100. Similar to the first classification task, performances of purely synthetically trained models were comparable to the ones from mixed training, especially for larger training set sizes, indicating that more synthetic data might be needed to achieve the same level of accuracy. Detailed results are listed in Supplementary Table 3.

### Prognosis of patients based on model predictions

**Fig. 5:**
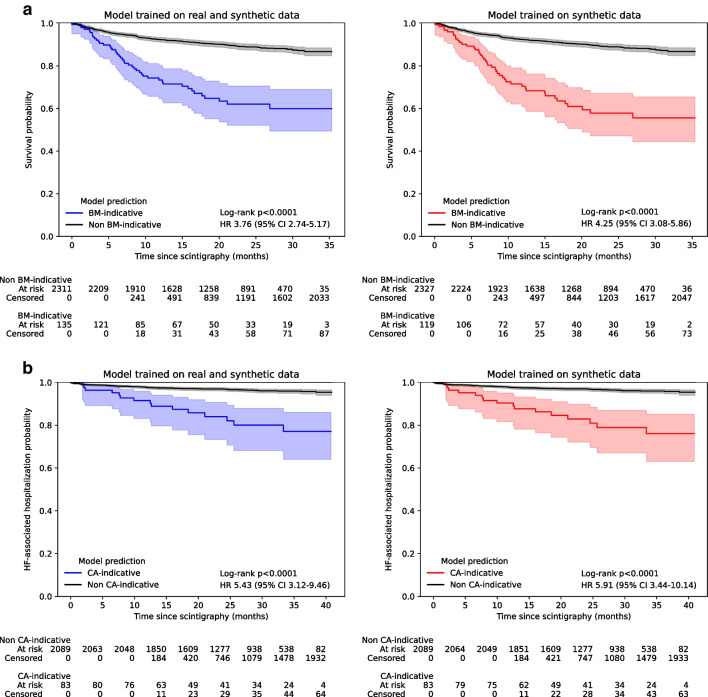
Kaplan-Meier estimates for patients in Cohort D stratified by the model predictions. **a** All-cause mortality was the endpoint for patients with predicted bone metastases-indicative tracer uptake. **b** Heart failure-associated hospitalization served as the endpoint for patients with predicted cardiac amyloidosis-indicative uptake

Outcome data was available for Cohort D, which included 2,446 patients. Data on all-cause mortality were available for all 2,446 patients (100%), of which 262 (11%) had died after a median follow-up of 1.8 years (IQR 1.1–2.4) after first scintigraphy. Predicted BM-indicative tracer uptake was significantly associated with all-cause mortality. This was true for a model trained on mixed (real and synthetic) data (crude HR 3.76 [2.74–5.17]; log-rank *p* < 0.0001; Fig. 5A first column) and a purely synthetically trained model (4.25 [3.08–5.86]; log-rank *p* < 0.0001; Fig. 5A second column). BM-indicative tracer uptake remained significantly associated with mortality after the multivariate adjustment for confounders (Supplemental Table 4), resulting in an adjusted HRs of 3.09 [2.24–4.26] (*p* < 0.0001) for the mixed model and 3.50 [2.53–4.85] (*p* < 0.0001) for the synthetic model.

Data on heart failure-associated hospitalization was available for 2,172 (89%) patients. After a median follow-up of 2.4 years (IQR 1.7–2.9) after scintigraphy, 83 (4%) of the 2,172 patients were hospitalized due to heart failure. Predicted CA-indicative uptake was a significant predictor for heart failure-associated hospitalization, irrespective of the model being trained on mixed data (crude HR 5.43 [95% CI 3.12–9.46]; log-rank *p* < 0.0001; Fig. 5B first column) or synthetic data only (5.91 [3.44–10.14]; log-rank *p* < 0.0001; Fig. 5B second column) and remained significant after multivariate adjustment (Supplemental Table 5) leading to adjusted HRs of 2.79 [1.56, 4.98] (*p* = 0.0005) for mixed and 3.05 [1.73–5.37] (*p* = 0.0001) for synthetic data.

The Kaplan-Meier estimates and hazard ratios for the reference standard (ground truth annotation) is shown in Supplemental Fig. 2.

## Discussion

The progress of deep learning in medical imaging is constrained by the lack of extensive, annotated datasets necessary for effectively training deep learning models. While collecting large datasets may be impractical or even impossible at smaller centers such as community hospitals, collecting data over multiple centers offers a viable alternative for enriching local databases. This becomes even more important for rare conditions that can be identified through bone scintigraphy, including but not limited to cardiac amyloidosis, Paget’s disease, and Osteoid Osteoma [25]. Here, the low prevalence in the general population makes the collection of representative datasets a lengthy process, even at large-scale centers. However, due to the significant challenges in sharing medical data across institutions, primarily due to patient privacy concerns, researchers often resort to using small, single-center datasets with limited diversity. Moreover, the associated annotation effort of large datasets is labor-intensive and requires expert input, which further complicates the data collection and curation process. These difficulties pose significant hurdles in developing reliable models and hamper the progress of clinical translation.

In response, we trained a generative model to create synthetic medical imaging data using bone scintigraphy as an example of an imaging modality in nuclear medicine. To our knowledge, this is the first study investigating the capabilities of generative models to create synthetic molecular imaging data for the development of downstream deep learning models. We evaluated whether synthetic data can complement small datasets to improve the development of deep learning models in limited data regimes. We used a comprehensive all-comer database of routinely acquired whole-body bone scintigraphy scans from a tertiary university hospital covering various pathologies and showed that the generative model successfully learned to synthesize scans from the underlying data distribution. Furthermore, we demonstrated the high quality of the AI-generated images, which were indistinguishable from real scintigraphy scans as assessed in a blinded reader study by four experts. We showcased that by controlling the data generation through conditioning variables, we were able to create authentic images related to predefined pathologies. Moreover, our analysis confirmed that the synthetic images are not mere replicas of the training data, thus ensuring patient privacy. Importantly, we also demonstrated that augmenting small datasets with synthetic images increased training data diversity and improved the performance of deep learning models in downstream classification tasks, such as detecting abnormal uptake indicative of bone metastases or cardiac amyloidosis. The performance increase was consistent across external centers when compared to the baseline, including various scanners and tracers, suggesting an improved generalization ability of the model through the added synthetic data without disclosing patient-sensitive information. The clinical significance of these findings was emphasized by revealing the associations between the model predictions and clinical outcomes. Our results suggest that classification models trained on synthetic bone scintigraphy images can lead to prognostic models and better performance without relying on real-world data once trained generative models are available.

The capabilities of generative models to synthesize medical imaging data have been studied for different imaging modalities. Schütte et al. conducted a comprehensive evaluation study about chest X-rays and 2D brain CT image generation [10]. Han et al. studied the generation of synthetic chest X-rays and investigated the potential of federated learning strategies for generative models [11]. Both studies found that the reader’s accuracy in discerning synthetic from real images improved by up to 80% with an increasing resolution. This poses a substantial limitation in comparison to the present study, as it indicates that their approaches failed to generate realistic images at conventional resolutions used in clinical practice. Most importantly, both studies performed their experiments on images representing lower resolutions compared with images employed in clinical routine, therefore substantially reducing their similarity to real-world medical images as well as their clinical applicability. A preliminary study in the field of molecular imaging used a stable diffusion model to generate synthetic thyroid scintigraphy images for enhancing deep learning-based multiclass thyroid classification. The authors reported improved model performance after incorporating synthetic data into the training process [26].

Our study is subject to several limitations. We used the anterior views of bone scintigraphy scans to train the generative model and the downstream classification model. This might have negatively impacted the obtained classification accuracy as certain pathologies might only be visible in the posterior projection. At one center (Champalimaud Foundation), a different protocol was used for annotating BM-indicative uptake compared to the standardized protocol followed by the other four centers, which could have impacted the performance results. Cohort D was used for external testing of the classification model. Since the generative model was trained on data (Cohort A) from the same hospital (Vienna General Hospital), adding synthetic data might qualify it as in-distribution data despite the two datasets originating from different time intervals (Cohort A: 2010–2020, Cohort D: 2020–2023). Hence, the results of Cohort D cohort should be interpreted more carefully compared to the other external testing cohorts. While our findings may be of interest to environments where data privacy concerns or data scarcity limits access to real datasets entirely, they should be considered cautiously. These findings do not imply that generated data can replace real data. Synthetic data, although proven useful in this study, might not always capture the complex distribution of real-world routine data, especially in cases that could be considered out of distribution, such as images with drains, tubes, or catheters. This limitation could lead to overfitted models when training is performed solely on synthetic data, underscoring the importance of evaluating models on large and diverse patient cohorts - including multiple centers, scanners, and tracers - as demonstrated in this study, to ensure robustness and generalizability. Although synthetic medical imaging data represents a promising alternative to develop generalizable deep learning models in low data regimes, it is important to note that generating high-quality synthetic data itself requires a large and well-curated dataset to train the generative model. Thus, the challenge of collecting comprehensive datasets remains a critical hurdle. Furthermore, imaging technology, protocols, or even diagnostic procedures are not static and may change over time, requiring generative models to be continuously updated with the latest data to prevent downstream models from becoming outdated. Since generative models learn from the underlying training distribution, biases in the original data may be preserved. Therefore, the use of synthetic data should be transparently reported, and downstream models should be rigorously tested with large, diverse evaluation cohorts. Finally, the question of how privacy guarantees can be provided when synthetic data is shared across institutions remains open. Although privacy assurance assessments can be performed, there is no definitive method to ensure that individual data points cannot be reconstructed or inferred from the synthetic data, for instance, when a synthetic image is composed of individual parts of multiple real images. Future studies investigating the robustness of privacy protection methods for synthetic data and their associated risks are essential to ensure that synthetic data can be safely shared across institutions without compromising patient confidentiality.

This study demonstrates the capabilities of generative AI to create high-quality bone scintigraphy images that accurately reflect prespecified pathologies. Incorporating synthetic images increased training data diversity and improved the performance of deep learning models in detecting disease-associated abnormal uptake. These improvements were consistent across multiple centers, scanners, and tracers, suggesting robust generalizability. The association between predicted abnormal uptake and adverse clinical outcomes underscores the validity and clinical relevance of our findings and highlights the promising role of synthetic data in advancing medical imaging research in data-constrained environments.

## Supplementary Information

Below is the link to the electronic supplementary material.

ESM 1 (DOCX 1.69 MB)

## Data Availability

A dataset of representative synthetic images is publicly available at 10.5281/zenodo.13275306.
