# Supplementary material for: Generative artificial intelligence enables the generation of bone scintigraphy images and improves generalization of deep learning models in data-constrained environments
Source: Eur J Nucl Med Mol Imaging. 2025 Jan 29;52(7):2355–68. doi: 10.1007/s00259-025-07091-8 (PMC12119683; doi:10.1007/s00259-025-07091-8)
Supplement: Supplementary file 1 — (DOCX 1.69 MB) [file 259_2025_7091_MOESM1_ESM.docx]

**SUPPLEMENTAL MATERIAL**

**Generative artificial intelligence enables the generation of bone scintigraphy images and improves generalization of deep learning models in data-constrained environments**

David Haberl^1,2^, Jing Ning^1,2^, Kilian Kluge^1,2^, Katarina Kumpf^3^, Josef Yu^1^, Zewen Jiang^1,2^, Claudia Constantino^4^, Alice Monaci^5^, Maria Starace^5^, Alexander R. Haug^1,2^, Raffaella Calabretta^1^, Luca Camoni^6^, Francesco Bertagna^6^, Katharina Mascherbauer^7^, Felix Hofer^7^, Domenico Albano^6^, Roberto Sciagra^5^, Francisco Oliveira^4^, Durval Costa^4^, Christian Nitsche^7^, Marcus Hacker^1^, Clemens P. Spielvogel^1^

^1^Department of Biomedical Imaging and Image-guided Therapy, Division of Nuclear Medicine, Medical University of Vienna, Vienna, Austria

^2^Christian Doppler Laboratory for Applied Metabolomics, Medical University of Vienna, Vienna, Austria

^3^IT4Science, IT Services & Strategic Information Management, Medical University of Vienna, Vienna, Austria

^4^Nuclear Medicine-Radiopharmacology, Champalimaud Clinical Centre, Champalimaud Foundation, Lisbon, Portugal

^5^Department of Experimental and Clinical Biomedical Sciences, Nuclear Medicine Unit, University of Florence, Florence, Italy

^6^ASST Spedali Civili of Brescia, Università degli Studi di Brescia, Brescia, Italy

^7^Department of Internal Medicine II, Division of Cardiology, Medical University of Vienna, Vienna, Austria

**A. Generative model**

Synthetic bone scintigraphy images were generated using a conditional StyleGAN2 architecture (*1*). StyleGAN2 encompasses two deep neural networks that are trained in a competitive manner (Supplemental Figure 5). A style-based generator network G is trained to generate synthetic bone scintigraphy scans. A discriminator network D is directly coupled to the output of G and trained to distinguish between synthetic and real images. Through this process, the generator G is iteratively improving at synthesizing images, eventually resulting in deceiving the discriminator by generating highly realistic-looking images that are indistinguishable from real images. It is important to note that only the discriminator network is trained with real patient images, while the generator, the network that is later used to create synthetic images, is never directly exposed to patient-sensitive data during the entire training process. The initial training length was set to two million images, i.e., training stops after the discriminator has seen this number of images. The model was periodically saved after 100,000 images, and the best-performing model was selected for inference.

**B. Privacy assurance assessment**

We used a pre-trained ResNet50 model to extract features from each real and synthetic image (*2*). The model was pre-trained on the ImageNet database (*3*). After feature extraction, each real and synthetic image was represented by a feature vector consisting of 2048 features. We used those feature vectors for the image similarity analysis as they are invariant to spatial locations in the original images. To visualize the training and synthetic data distribution in a 2D scatterplot, we further reduced the 2048 features to 2 features using the uniform manifold approximation and projection (UMAP) dimensionality reduction technique (*4*).

**C. Deep learning model and downstream classification**

A convolutional neural network based on the DenseNet121 architecture (*5*) was used. The model was pre-trained on the ImageNet database (*3*). The model’s weights were fine-tuned on the dedicated target dataset using the Adam optimizer with a constant learning rate of 0.00001 and a weight decay of 0.001. The initial training length was set to 100 epochs in combination with a validation-based early stopping configuration of 15 tolerance epochs and a minimum change of loss of 0. The batch size was set to 64. Standard data augmentations (rotations, zooms, additive noise, shifted and scaled intensities, smoothing) were performed on the training set. Random oversampling of the minority class was applied during training. The same model architecture, training scheme, and hyperparameters were used for both classification tasks.

**Supplemental Fig. 1** Fréchet inception distance (FID) scores of the GAN training process for (a) generating images with uptake indicative of bone metastases and (b) uptake indicative of cardiac amyloidosis. After processing 600,000 images during training the best-performing model achieved an FID of 28.94 for synthesizing scans with or without BM-indicative uptake and an FID of 9.91 for scans with or without CA-indicative uptake


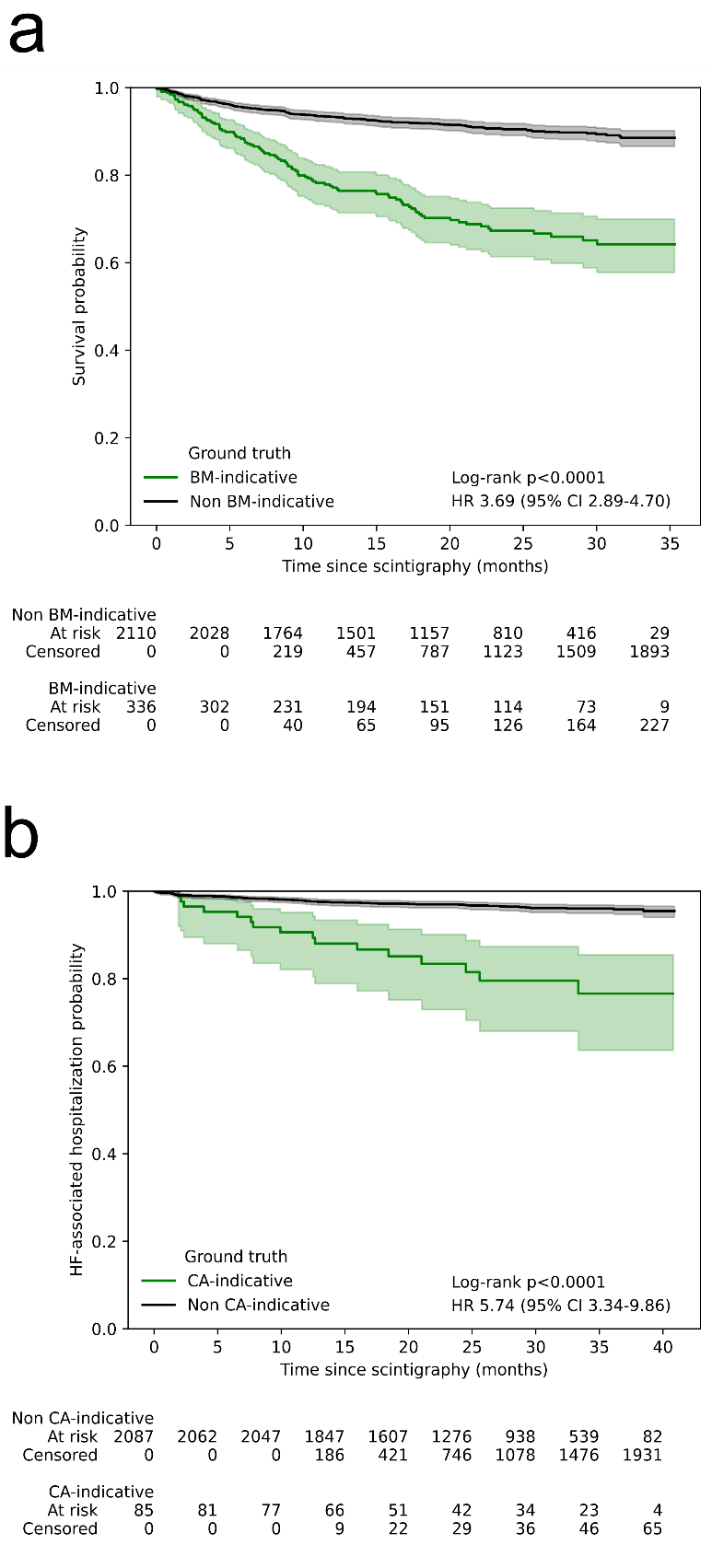


**Supplemental Fig. 2** Kaplan-Meier estimates for patients in Cohort D stratified by the ground truth annotations. (a) All-cause mortality was the endpoint for patients with annotated bone metastases-indicative tracer uptake. (b) Heart failure-associated hospitalization served as the endpoint for patients with annotated cardiac amyloidosis-indicative uptake


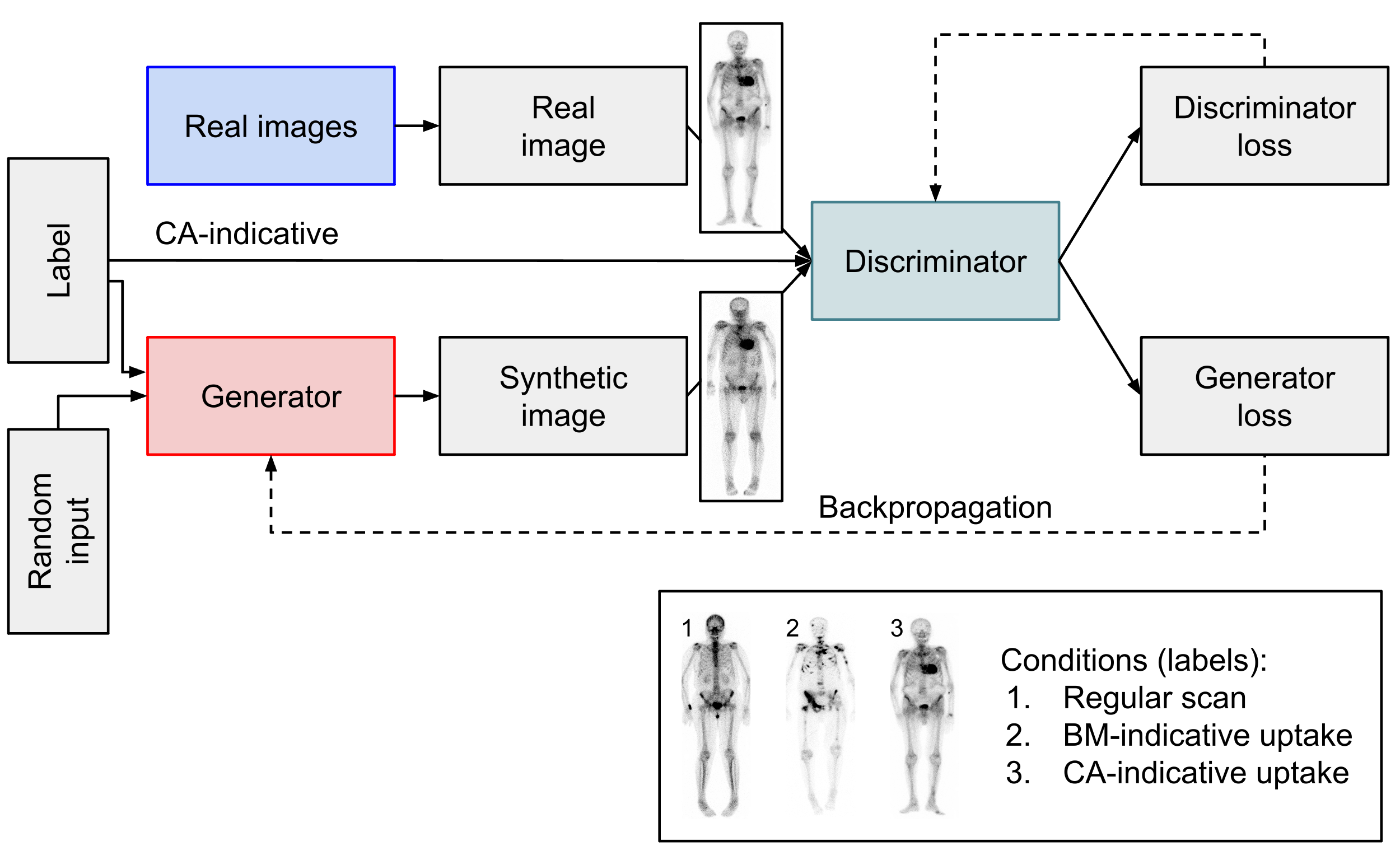


**Supplemental Fig. 3** Architecture and training scheme of the conditional generative adversarial network

**Supplemental Table 1** Acquisition characteristics

| **Cohort** | **Tracer** | **Scanner** |
| --- | --- | --- |
| Vienna General Hospital,Cohort A | DPD | Discovery 670 hybrid gamma camera (GE HealthCare Technologies, Chicago, IL, USA)  Varicam (GE HealthCare Technologies, Chicago, IL, USA)  IRIX (Koninklijke Philips, Amsterdam, Netherlands)  Encore2 (Siemens Healthineers, Erlangen, Germany)  Symbia Intevo (Siemens Healthineers, Erlangen, Germany)  Tandem 870 CZT (GE HealthCare Technologies, Chicago, IL, USA) |
| ASST Spedali Civili of Brescia, Cohort B | DPD | Infinia (GE HealthCare Technologies, Chicago, IL, USA)  Tandem Discovery 670 Pro (GE HealthCare Technologies, Chicago, IL, USA) |
| Careggi University Hospital, Cohort C | HMDP | Discovery 630 (GE HealthCare Technologies, Chicago, IL, USA) |
| Vienna General Hospital, Cohort D | DPD | Encore2 (Siemens Healthineers, Erlangen, Germany)  Symbia Intevo (Siemens Healthineers, Erlangen, Germany)  Tandem 870 CZT (GE HealthCare Technologies, Chicago, IL, USA) |
| Champalimaud Foundation, Cohort E | HMDP | BrightView (Koninklijke Philips, Amsterdam, Netherlands) |
| West China Hospital, Cohort F | Not reported | Not reported |

**Supplemental Table 2** Model performances for the prediction of tracer uptake indicative of bone metastases. Values are the area under the receiver-operating characteristic curve and 95% confidence intervals. The best performing models were consistently associated with a significantly increased AUC when compared to the baseline (p<0.0001)

|  | **Training set size** | | | | | | | |
| --- | --- | --- | --- | --- | --- | --- | --- | --- |
|  | n=181 | n=362 | n=543 | n=1086 | n=1991 | n=4706 | n=9231 | n=18281 |
| Real-to-synthetic data ratio | 1:0 | 1:1 | 1:2 | 1:5 | 1:10 | 1:25 | 1:50 | 1:100 |
| Cohort B | 0.436  (0.251-0.639) | 0.700  (0.580-0.813) | 0.549  (0.373-0.735) | 0.695  (0.498-0.863) | 0.868  (0.676-0.986) | 0.917  (0.801-0.984) | 0.927  (0.802-0.997) | 0.829  (0.630-0.992) |
| Cohort C | 0.487  (0.333-0.647) | 0.586  (0.409-0.746) | 0.702  (0.535-0.842) | 0.814  (0.686-0.925) | 0.823  (0.677-0.942) | 0.858  (0.745-0.954) | 0.895  (0.813-0.963) | 0.896  (0.812-0.960) |
| Cohort D | 0.618  (0.590-0.644) | 0.732  (0.709-0.756) | 0.774  (0.750-0.798) | 0.821  (0.800-0.842) | 0.866  (0.848-0.883) | 0.881  (0.864-0.898) | 0.895  (0.879-0.909) | 0.879  (0.861-0.896) |
| Cohort E | 0.544  (0.500-0.592) | 0.637  (0.592-0.681) | 0.683  (0.640-0.725) | 0.742  (0.703-0.783) | 0.755  (0.713-0.791) | 0.778  (0.736-0.814) | 0.789  (0.751-0.827) | 0.778  (0.735-0.816) |
| Cohort F | 0.626  (0.590-0.662) | 0.674  (0.636-0.712) | 0.686  (0.649-0.721) | 0.736  (0.699-0.768) | 0.787  (0.755-0.819) | 0.840  (0.807-0.868) | 0.851  (0.822-0.879) | 0.840  (0.810-0.870) |
| Real-to-synthetic data ratio | 0:1 | 0:2 | 0:3 | 0:6 | 0:11 | 0:26 | 0:51 | 0:101 |
| Cohort B | 0.676  (0.539-0.800) | 0.736  (0.585-0.874) | 0.853  (0.747-0.936) | 0.832  (0.717-0.926) | 0.942  (0.874-0.990) | 0.936  (0.849-0.996) | 0.980  (0.941-1.000) | 0.973  (0.935-1.000) |
| Cohort C | 0.574  (0.426-0.725) | 0.601  (0.467-0.726) | 0.753  (0.615-0.886) | 0.808  (0.669-0.928) | 0.823  (0.687-0.937) | 0.801  (0.662-0.922) | 0.833  (0.717-0.923) | 0.879  (0.801-0.945) |
| Cohort D | 0.737  (0.714-0.759) | 0.755  (0.735-0.777) | 0.809  (0.788-0.829) | 0.803  (0.782-0.822) | 0.875  (0.859-0.891) | 0.877  (0.859-0.893) | 0.874  (0.855-0.892) | 0.881  (0.863-0.897) |
| Cohort E | 0.659  (0.617-0.698) | 0.706  (0.665-0.747) | 0.739  (0.699-0.779) | 0.743  (0.702-0.781) | 0.781  (0.744-0.819) | 0.773  (0.735-0.811) | 0.777  (0.735-0.818) | 0.777  (0.733-0.816) |
| Cohort F | 0.677  (0.636-0.718) | 0.663  (0.627-0.700) | 0.661  (0.625-0.695) | 0.689  (0.650-0.726) | 0.814  (0.782-0.846) | 0.827  (0.794-0.856) | 0.857  (0.826-0.885) | 0.829  (0.798-0.858) |

**Supplemental Table 3** Model performances for the prediction of cardiac uptake indicative of cardiac amyloidosis. Values are the area under the receiver-operating characteristic curve and 95% confidence intervals. The best performing models were consistently associated with a significantly increased AUC when compared to the baseline (p<0.0001)

|  | **Training set size** | | | | | | | |
| --- | --- | --- | --- | --- | --- | --- | --- | --- |
|  | n=181 | n=362 | n=543 | n=1086 | n=1991 | n=4706 | n=9231 | n=18281 |
| Real-to-synthetic data ratio | 1:0 | 1:1 | 1:2 | 1:5 | 1:10 | 1:25 | 1:50 | 1:100 |
| Cohort B | 0.900  (0.850-0.942) | 0.988  (0.972-0.998) | 0.997  (0.992-1.000) | 0.982  (0.960-0.997) | 0.989  (0.964-1.000) | 0.989  (0.972-1.000) | 0.994  (0.982-1.000) | 0.999  (0.997-1.000) |
| Cohort C | 0.968  (0.946-0.986) | 0.987  (0.976-0.996) | 0.992  (0.982-0.998) | 0.993  (0.985-0.999) | 1.000  (0.998-1.000) | 1.000  (0.999-1.000) | 1.000  (0.998-1.000) | 1.000  (0.999-1.000) |
| Cohort D | 0.978  (0.961-0.992) | 0.991  (0.984-0.996) | 0.991  (0.984-0.996) | 0.993  (0.987-0.998) | 0.997  (0.993-0.999) | 0.997  (0.995-0.999) | 0.997  (0.993-0.999) | 0.997  (0.991-0.999) |
| Real-to-synthetic data ratio | 0:1 | 0:2 | 0:3 | 0:6 | 0:11 | 0:26 | 0:51 | 0:101 |
| Cohort B | 0.956  (0.927-0.979) | 0.949  (0.915-0.975) | 0.986  (0.971-0.996) | 0.998  (0.995-1.000) | 0.998  (0.994-1.000) | 0.997  (0.991-1.000) | 0.999  (0.995-1.000) | 0.999  (0.997-1.000) |
| Cohort C | 0.836  (0.776-0.891) | 0.842  (0.788-0.891) | 0.922  (0.884-0.956) | 0.974  (0.955-0.988) | 0.989  (0.976-0.997) | 1.000  (1.000-1.000) | 0.998  (0.994-1.000) | 1.000  (1.000-1.000) |
| Cohort D | 0.964  (0.947-0.979) | 0.964  (0.949-0.977) | 0.980  (0.968-0.990) | 0.990  (0.984-0.996) | 0.991  (0.983-0.996) | 0.997  (0.995-0.999) | 0.997  (0.994-0.999) | 0.998  (0.996-0.999) |

**Supplemental Table 4** Results of the Cox regression analysis for evaluating the association of parameters with mortality for the prediction of uptake indicative of bone metastases

|  |  | **Univariate** | | **Multivariate** | |
| --- | --- | --- | --- | --- | --- |
| **Model trained on** | **Parameter** | **Hazard ratio (95% CI)** | **p-value** | **Hazard ratio (95% CI)** | **p-value** |
| Real and synthetic data | Model prediction | 3.76 (2.74-5.17) | <0.0001 | 3.09 (2.24-4.26) | <0.0001 |
|  | Age | 1.03 (1.02-1.04) | <0.0001 | 1.03 (1.02-1.04) | <0.0001 |
|  | Sex, male | 1.67 (1.31-2.13) | <0.0001 | 1.36 (1.06-1.75) | 0.0148 |
|  | Cardiac amyloidosis | 1.51 (0.93-2.47) | 0.0971 | - | - |
| Synthetic data | Model prediction | 4.25 (3.08-5.86) | <0.0001 | 3.50 (2.53-4.85) | <0.0001 |
|  | Age | 1.03 (1.02-1.04) | <0.0001 | 1.03 (1.02-1.04) | <0.0001 |
|  | Sex, male | 1.67 (1.31-2.13) | <0.0001 | 1.33 (1.04-1.71) | 0.0256 |
|  | Cardiac amyloidosis | 1.51 (0.93-2.47) | 0.0971 | - | - |

CI = Confidence interval

**Supplemental Table 5** Results of the Cox regression analysis for evaluating the association of parameters with heart failure-associated hospitalization for the prediction of uptake indicative of cardiac amyloidosis

|  |  | **Univariate** | | **Multivariate** | |
| --- | --- | --- | --- | --- | --- |
| **Model trained on** | **Parameter** | **Hazard ratio (95% CI)** | **p-value** | **Hazard ratio (95% CI)** | **p-value** |
| Real and synthetic data | Model prediction | 5.43 (3.12-9.46) | <0.0001 | 2.79 (1.56-4.98) | 0.0005 |
|  | Age | 1.08 (1.05-1.10) | <0.0001 | 1.07 (1.05-1.09) | <0.0001 |
|  | Sex, male | 1.30 (0.85-1.98) | 0.2288 | - | - |
| Synthetic data | Model prediction | 5.91 (3.44-10.14) | <0.0001 | 3.05 (1.73-5.37) | 0.0001 |
|  | Age | 1.08 (1.05-1.10) | <0.0001 | 1.07 (1.04-1.09) | <0.0001 |
|  | Sex, male | 1.30 (0.85-1.98) | 0.2288 | - | - |

CI = Confidence interval

**REFERENCES**

1. Karras T, Aittala M, Hellsten J, Laine S, Lehtinen J, Aila T. Training Generative Adversarial Networks with Limited Data. *arXiv [csCV]*. June 2020.

2. He K, Zhang X, Ren S, Sun J. Deep Residual Learning for Image Recognition. *arXiv [csCV]*. December 2015.

3. Deng J, Dong W, Socher R, Li L-J, Li K, Fei-Fei L. ImageNet: A large-scale hierarchical image database. In: 2009 IEEE Conference on Computer Vision and Pattern Recognition. IEEE; 2009:248-255.

4. McInnes L, Healy J, Melville J. UMAP: Uniform Manifold Approximation and Projection for Dimension Reduction. *arXiv [statML]*. February 2018.

5. Huang G, Liu Z, van der Maaten L, Weinberger KQ. Densely Connected Convolutional Networks. *arXiv [csCV]*. August 2016.
